# Frequency of apnea, bradycardia, and desaturations following first diphtheria-tetanus-pertussis-inactivated polio-*Haemophilus influenzae *type B immunization in hospitalized preterm infants

**DOI:** 10.1186/1471-2431-6-20

**Published:** 2006-06-19

**Authors:** Jackie Lee, Joan L Robinson, Donald W Spady

**Affiliations:** 1 Stollery Children's Hospital and Department of Pediatrics, University of Alberta, Edmonton, Alberta, Canada

## Abstract

**Background:**

Adverse cardiorespiratory events including apnea, bradycardia, and desaturations have been described following administration of the first diphtheria-tetanus-pertussis-inactivated polio-*Haemophilus influenzae *type B (DTP-IPV-Hib) immunization to preterm infants. The effect of the recent substitution of acellular pertussis vaccine for whole cell pertussis vaccine on the frequency of these events requires further study.

**Methods:**

Infants with gestational age of ≤ 32 weeks who received their first DTP-IPV-Hib immunization prior to discharge from two Edmonton Neonatal Intensive Care Units January 1, 1996 to November 30, 2000 were eligible for the study. Each immunized infant was matched by gestational age to one control infant. The number of episodes of apnea, bradycardia, and/or desaturations (ABD) and the treatment required for these episodes in the 72 hours prior to and 72 hours post-immunization (for the immunized cohort) or at the same post-natal age (for controls) was recorded.

**Results:**

Thirty-four infants who received DTP-IPV-Hib with whole cell pertussis vaccine, 90 infants who received DTP-IPV-Hib with acellular pertussis vaccine, and 124 control infants were entered in the study. Fifty-six immunized infants (45.1%) and 36 control infants (29.0%) had a resurgence of or increased ABD in the 72 hours post-immunization in the immunized infants and at the same post-natal age in the controls with an adjusted odds ratio for immunized infants of 2.41 (95% CI 1.29,4.51) as compared to control infants. The incidence of an increase in adverse cardiorespiratory events post-immunization was the same in infants receiving whole cell or acellular pertussis vaccine (44.1% versus 45.6%). Eighteen immunized infants (14.5%) and 51 control infants (41.1%) had a reduction in ABD in the 72 hours post- immunization or at the equivalent postnatal age in controls for an odds ratio of 0.175 (95%CI 0.08, 0.39). The need for therapy of ABD in the immunized infants was not statistically different from the control infants. Lower weight at the time of immunization was a risk factor for a resurgence of or increased ABD post-immunization. Birth weight, gestational age, postnatal age or sex were not risk factors.

**Conclusion:**

There is an increase in adverse cardiorespiratory events following the first dose of DTP-IPV-Hib in preterm infants. Lower current weight was identified as a risk factor, with the risk being equivalent for whole cell versus acellular pertussis vaccine. Although most of these events are of limited clinical significance, cardiorespiratory monitoring of infants who are sufficiently preterm that they are receiving their first immunization prior to hospital discharge should be considered for 72 hours post-immunization.

## Background

The current recommendation is to administer the first routine immunization to preterm infants at the usual chronological age of 8 weeks as the immunologic response to vaccines at that age is almost equivalent to that of term infants [1]. However, in multiple previous studies, a 7 to 47% incidence of adverse cardiorespiratory events including apnea, bradycardia, and/or desaturation (ABD) has been reported following the first diphtheria-tetanus-pertussis-inactivated polio-*Haemophilus influenzae *type B (DTP-IPV-Hib) immunization of preterm infants [2-10], with the most common theory being that these events are precipitated by the whole cell pertussis component of the vaccine [3]. Previous studies used infants as their own controls, comparing events prior to and following immunization. Birth weight, current weight, gestational age, and postnatal age have been studied as potential risk factors for ABD with inconclusive results [4-7,9,10].

A retrospective stratified cohort study was completed to determine the frequency of adverse cardiorespiratory events among premature infants following their first immunization with DTP-IPV-Hib vaccine and to compare the frequency of events following whole cell versus acellular pertussis vaccines. A further objective was to study possible risk factors for these adverse events.

## Methods

Approval from the Health Research Ethics Board of the University of Alberta was obtained for conducting this study.

### Patients

Infants with a gestational age of ≤ 32 weeks who received their first DTP-IPV-Hib immunization prior to discharge from two Neonatal Intensive Care Units (NICUs) at the University of Alberta and Royal Alexandra Hospitals in Edmonton, Alberta January 1, 1996 to November 30, 2000 were eligible for the study. A public health nurse visits each NICU on a frequent basis and administers immunizations to all infants who have reached 8 weeks of age, assuming the attending physician identifies no contraindication. Compliance with this intervention is excellent with almost all infants discharged after 8 weeks of age having received DTP-IPV-Hib. Each immunized infant was then matched by gestational age to one control selected from infants not yet discharged from NICU at an equivalent chronologic age (some of whom had already been immunized) during the 5-year study period by using a random numbers table. The study date for controls was equivalent to the postnatal age at time of immunization of the matched immunized infant, with the date being adjusted up to seven days in either direction to avoid the control's own immunization, discharge, septic work up (as infants unlikely to be immunized within seven days of a septic work) or surgical procedure. Infants could be entered in the study once as a control and once as an immunized infant, spanning different dates each time. Immunized infants and controls were excluded if they were not on a cardiorespiratory monitor in the NICU for the entire observation period, they were still ventilated, there was concurrent administration of other vaccines, or documentation of case notes was inadequate.

### Definition of adverse cardiorespiratory events

The frequency of ABD was recorded in two observation periods for each infant. For immunized infants, observation period #1 was the 72 hours pre-immunization and observation period #2 was the 72 hours post-immunization. For controls, observation period #1 was the 72 hours prior to the date that corresponded to the equivalent age at which the infant they were matched to was immunized and observation period #2 was that date and the 48 hours following that date. ABD were detected by cardiorespiratory monitors or by observation and recorded by nurses caring for the infants. Apnea was defined as cessation of respiration for 20 seconds or more. Bradycardia was defined as a heart rate less than 100 beats per minute for 20 seconds or more. Desaturation was defined as oxygen saturation ≤ 85%. An increase in adverse cardiorespiratory event was defined as: 1) a resurgence of ABD as indicated by greater than one ABD within observation period #2 with no ABD during observation period #1, or 2) increased ABD as indicated by a 25% increase in the number of ABD events within observation period #2 versus observation period #1. A reduction in adverse cardiorespiratory events was defined as: 1) resolution of ABD as indicated by greater than one ABD event in observation period #1 with none in observation period #2 or 2) decreased ABD as indicated by a 25% decrease in ABD events within observation period #2 versus observation period #1.

### Data collection

Birth weight, current weight, gestational age, sex, and theophylline or doxapram use were recorded for immunized infants and for controls, and the postnatal age at the time of immunization and the vaccine type were recorded for immunized infants. The number of episodes of ABD and the treatment required for these events in observation periods #1 and #2 were recorded for immunized infants and for controls.

### Statistical analysis

Data were analyzed using Stata. Cellular and acellular vaccines were compared by using Chi-square. A p < 0.05 was considered statistically significant. Odds ratios were calculated using conditional logistic regression with the dependent variable being either (a) an increase in adverse cardiorespiratory events or (b) a decrease in adverse cardiorespiratory events and the independent variable being immunization. Differences were considered statistically significant if the 95% confidence interval of the odds ratio did not include the value one.

## Results

### Patients

One hundred ninety-two infants received their first DTP-IPV-Hib immunization in NICU during the study period. DTP-IPV-Hib with whole cell pertussis vaccine was used prior to July 1, 1997 and DTP-IPV-Hib with acellular pertussis vaccine was used starting on this date. Sixty infants had exclusion criteria and a further eight immunized infants had to be excluded as an appropriate control infant could not be identified.

### Patient characteristics

One hundred twenty-four immunized infants and 124 controls were evaluated. The study date for controls was adjusted up to seven days in either direction to avoid the control's own immunization (n = 35), discharge (n = 10), septic work up (n = 9), or surgical procedure (n = 1). There was no statistical difference between the immunized infants and controls in birth weight, current weight, gestational age, postnatal age or sex (Table 1). The mean birth weight of immunized infants was 893 ± 236 grams (range 110–1730 grams), the mean current weight was 2056 ± 510 grams (range 1049–3220 grams), the mean gestational age was 26.5 ± 1.7 weeks (range 23–31 weeks), and 58% were male. The postnatal age of the infants at immunization was < 70 days (n = 66), 70 to 100 days (n = 50), 101 to 129 days (n = 7) and > 120 days (n = 1) for a mean age of 74 ± 14 days (range 58–125 days). Thirty-four infants received DTP-IPV-Hib with whole cell pertussis vaccine and 90 received DTP-IPV-Hib with acellular pertussis vaccine.

**Table 1:** Comparison of preterm infants receiving their first DTP-IPV-Hib immunization to control infants.

	Immunized infants (n = 124)	Controls (n = 124)
Birth weight (grams)	893 (range 110–1730)	892 (range 110–1840)
Weight at immunization (grams)	2056 (range 1049–3220)	2085 (range 1054–3500)
Gestational age (weeks)	26 (range 23–31)	26 (range 23–32)
Postnatal age at immunization (days)	74 (range 58–125)	74 (range 56–120)
Male sex	58%	60%

### Incidence of adverse cardiorespiratory events

Fifty-six (45.1%) of the 124 infants who received their first DTP-IPV-Hib and 36 (29.0%) of the 124 control infants had an increase in adverse cardiorespiratory events during observation period #2 (Table 2). There was a statistically significant difference in the incidence of increase in adverse cardiorespiratory events between immunized infants and controls with an adjusted odds ratio for immunized infants of 2.41 (95% CI 1.3, 4.5). Fifteen of the 34 infants (44.1%) who received DTP-IPV-Hib with whole cell pertussis vaccine had an increase in adverse cardiorespiratory events post-immunization, as compared to 41 of the 90 infants (45.6%) who received DTP-IPV-Hib with acellular pertussis vaccine (χ^2 ^0.021; p = 0.89). Eighteen (14.5%) of the 124 infants who received their first DTP-IPV-Hib had a reduction in cardiorespiratory events in observation period #2 compared to 51 (41.1%) of the 124 control infants (odds ratio 0.175; 95% CI 0.08, 0.39).

**Table 2:** Incidence of adverse cardiorespiratory events in preterm infants in the 72 hours following first immunization with DTP-IPV-Hib vaccine as compared to control infants.

	Immunized infants (n = 124)	Controls (n = 124)	OR*	95% CI
Increase in adverse cardiorespiratory events	56	36	2.41	1.3,4.5
Resurgence of ABD	9	2	4.50	0.97,20.8
Increase in ABD	47	34	1.60	0.94,2.80

Reduction in adverse cardiorespiratory events	18	51	0.18	0.08,0.39
Resolution of ABD	6	4	1.7	0.40,6.90
Decrease in ABD	12	47	0.13	0.05,0.32

* adjusted odds ratio for immunization relative to no immunization

Of the 56 immunized infants with adverse cardiorespiratory events, 9 infants (16.1%) had increased oxygen requirements and/or required initiation of continuous positive airway pressure (CPAP) and 9 infants (16.1%) required an increased theophylline dose. A 26-week gestation male infant who received acellular pertussis vaccine required mechanical ventilation on the third day post-immunization as he had a 36% increase in apneas post-immunization that persisted despite initiation of CPAP and theophylline. The need for therapy of adverse cardiorespiratory events in the immunized infants was not statistically different from the control group where 6 infants (16.6%) had increased oxygen requirements and/or required initiation of CPAP and 7 infants (19.4%) required an increased theophylline dose.

Lower weight at the time of immunization was a risk factor for an increase in adverse cardiorespiratory events, with the odds ratio per 100 gram increase in weight being 0.83 (95% CI 0.72, 0.96). There was no statistically significant difference in birth weight, gestational age, postnatal age or sex in infants with and without an increase in adverse cardiorespiratory events post-immunization.

## Discussion

Previous studies have identified a possible increase in the frequency of adverse cardiorespiratory events following the first DTP-IPV-Hib immunization in preterm infants [2-10]. Our study was the first to use a control group and confirmed that in infants ≤ 32 weeks gestation, almost half of infants had an increase of adverse cardiorespiratory events in the 72 hours post-immunization which was statistically significantly higher than in the control group. Furthermore, the number of immunized infants with a reduction in adverse cardiorespiratory events in the 72 hours post-immunization was statistically significantly lower than in the control group at an equivalent chronologic age, and an immunized infant was only 17% as likely as a control infant to have a reduction in adverse cardiorespiratory events. Comparing our adverse event rate to those reported in the literature, one small prospective study found no change in the frequency of apnea or increased oxygen requirements in 16 infants < 29 weeks gestation receiving whole cell pertussis vaccine [11], while other studies reported an incidence of adverse cardiorespiratory events of only ~8% in infants receiving whole cell pertussis vaccine [2,3]. Also using whole cell pertussis vaccine, a retrospective study of 97 infants [4] and a follow up prospective study [5] by the same group found rates of adverse cardiorespiratory events with incidences of 20% and 17% respectively. Unexpectedly, higher incidences of 38 to 47% that are more comparable to the rate in the current study have been documented in more recent studies with acellular pertussis vaccines [8-10]. However, these varying rates may be partially accounted for by differences in the definition of cardiorespiratory events and in monitoring practices.

Despite the fact that the current study demonstrated an increase in adverse cardiorespiratory events post-immunization, most of these events were clinically insignificant and the number requiring treatment was not statistically different between the immunized and control groups. As described in the previous literature, most events resolved spontaneously or required only brief stimulation or transient low flow oxygen [3-10]. Sixteen percent of the immunized infants in the current study required increased oxygen or CPAP, which fits with the wide range of 0–33% reported in previous studies [3-10], while 16% required increased theophylline doses and a single infant required reintubation on the third day post-immunization. The relationship of this event to the immunization is not clear.

The main reason for changing from whole cell to acellular pertussis vaccine is the decreased reactogenicity of the latter vaccine. However, in the current study there was no statistically significant difference in the frequency of adverse cardiorespiratory events post-immunization with whole cell as compared to acellular pertussis vaccines in preterm infants. This differs from a previous study in preterm infants where increased adverse cardiorespiratory events and increased systemic inflammatory markers (IL-6, CRP) occurred in 30% of infants receiving whole cell pertussis vaccine but in no infants receiving acellular pertussis vaccine [7].

Previous studies found that the risk of adverse cardiorespiratory events post- immunization was higher in infants with lower birth weight [6], lower current weight [6], lower gestational age [4], or postnatal age < 70 days [10], while other studies did not confirm these findings [5,7] or found a correlation with the presence of adverse cardiorespiratory events during the 24 hours preceding the immunization [9]. In the current study, only the current weight appeared to be a risk factor where the infant's likelihood of having an adverse cardiorespiratory event decreased by 17% for every 100 gram increase in weight. Other potential risk factors such as chronic lung disease, duration of ventilation or oxygen dependency were not addressed in our study.

One limitation of our study is that patients not on electronic cardiorespiratory monitors 72 hours prior to and post-immunization were excluded. These infants would presumably be more stable and may be less likely to have an adverse event precipitated by the immunization. Therefore, the frequency of apnea or bradycardia in preterm infants receiving their first DTP-IPV- Hib immunization may have been over-estimated in the current study. The study dates of the controls that corresponded to the immunization date of the immunized infants were adjusted up to 7 days in 55 patients to avoid confounding factors, which could have affected results. It would have been ideal but impractical to use a shorter window. Charting of adverse cardiorespiratory events by nursing staff can vary and it is possible that nurses were more diligent about observing infants who had recently received an immunization. However, we suspect there was limited awareness amongst nurses or physicians of the potential for immunizations to precipitate adverse cardiorespiratory events, as many infants could not be included in the study as they were not on a monitor post-immunization. It has been suggested that when looking for adverse events related to immunization, it may be inappropriate to use unimmunized children as controls as they may differ in important ways from immunized children [12]. However, almost all control infants in the current study were already immunized or were eventually immunized. Because immunization is often arranged just prior to discharge, if there was a systemic bias, it would be towards the immunized infants being "more stable" than the control infants and less likely to have adverse cardiorespiratory events at this chronologic age.

## Conclusion

More hospitalized preterm infants ≤ 32 weeks developed an increase in adverse cardiorespiratory events after their first immunization with DTP-IPV-Hib than did a control group at an equivalent chronologic age. A lower weight at the time of immunization was a risk factor for an increase in adverse cardiorespiratory events. The risk of these events does not appear to have been decreased by the use of acellular pertussis vaccine. The majority of these events were clinically insignificant and should not prompt delays in immunization. However, whenever practical, immunization should be given at least 72 hours prior to planned discharge in this fragile population to allow for cardiorespiratory monitoring post-immunization.

## Competing interests

The author(s) declare that they have no competing interests.

## Authors' contributions

JL and JLR designed the study and wrote the manuscript. JL collected the data. DWS performed the statistical analysis and reviewed the manuscript.

## Pre-publication history

The pre-publication history for this paper can be accessed here:


